# Robust environmental exposure pattern recognition and outlier detection with the open-source R package pcpr

**DOI:** 10.1097/EE9.0000000000000515

**Published:** 2026-08-10

**Authors:** Lawrence G. Chillrud, Jaime Benavides, Elizabeth A. Gibson, Junhui Zhang, Jingkai Yan, John N. Wright, Jeff Goldsmith, Marianthi-Anna Kioumourtzoglou

**Affiliations:** a Department of Environmental Health Sciences, Columbia University Mailman School of Public Health, New York, New York; b Department of Electrical and Computer Engineering, Northwestern University, Evanston, Illinois; c Department of Epidemiology, Brown University School of Public Health, Providence, Rhode Island; d Department of Biostatistics, Harvard T. H. Chan School of Public Health, Boston, Massachusetts; e Department of Operations Research, MIT, Cambridge, Massachusetts; f Department of Electrical Engineering, Columbia University, New York, New York; g Department of Biostatistics, Columbia University Mailman School of Public Health, New York, New York; h Institute at Brown University for Environment and Society, Providence, Rhode Island

**Keywords:** Mixtures methods, Pattern recognition, Software, Dimensionality reduction

## Abstract

**Background:**

Pattern recognition in high-dimensional mixture data is of increasing interest in environmental health (EH), as researchers often aim to identify sources or behaviors leading to potentially harmful exposures. Principal component pursuit (PCP)—a robust dimensionality reduction technique—has been successfully utilized for pattern recognition in a number of EH studies. PCP decomposes an exposure matrix into a low-rank matrix encoding consistent exposure patterns and a sparse matrix isolating outlying exposure events. However, PCP’s application has been hindered by a lack of available software tailored specifically to EH research.

**Methods:**

We introduce an open-source R package, pcpr, enabling easy PCP deployment in EH research. The package provides functions to fit and fine-tune PCP models with three EH-specific extensions: (1) a non-negativity constraint on the low-rank matrix, enhancing interpretability; (2) procedures to accommodate missingness; and (3) a specialized penalty for observations below the analytic limit of detection. We illustrate core functionality by conducting a fully reproducible source-apportionment analysis of an air pollution mixture of 26 PM_2.5_ constituents measured every few days in Queens, New York City (2015–2021).

**Results:**

The functions in pcpr autonomously extracted four consistent exposure patterns (secondary signal and tailpipe emissions, traffic, crustal dust, and salt) and 211 outlying exposure events (including July Fourth firework-related spikes) from the Queens PM_2.5_ mixture. If found to be associated with adverse health outcomes in downstream health models, these exposure patterns and extreme events can support targeted regulatory action.

**Conclusion:**

The pcpr package facilitates robust, reproducible, and accessible exposure pattern recognition tailored to environmental epidemiology.

**What this study adds**

Traditional exposure pattern recognition methods, such as principal components analysis, are sensitive to outliers, noise, missing observations, and the analyst’s choice in the number of patterns to extract, limiting their utility and reproducibility in environmental health research. Moreover, a lack of standardized software tailored for work with environmental data hampers reproducibility even further. We developed an open-source R package implementing principal component pursuit, enabling robust and autonomous extraction of exposure patterns as well as outlying exposure events. A reproducible and accessible tool tailor-made for epidemiologic research, this software advances the ability to characterize complex environmental mixtures data in health studies.

## Introduction

Understanding the health effects of high-dimensional, complex mixtures data is of increasing interest in environmental epidemiology.^1–5^ While traditional single-chemical analyses examining exposure–response relationships have identified toxins and yielded important regulatory action, they fail to accurately reflect reality^5–7^; at any given time, humans are exposed to mixtures with thousands of chemical constituents. To capture the joint effects across these exposures, studies must assess exposure to multiple chemicals simultaneously.^6–9^

Exposure pattern recognition is an important tool for dealing with the technical challenges that come with analyzing multiexposure data.^6,10–12^ High-dimensional environmental mixtures are often characterized by severe multicollinearity and noisy, incomplete, or outlying observations (throughout the article, “noise” refers to the random error inherent to the data-generating process, e.g., imperfect air monitors injecting some amount of random error into air pollution measurements, while “incomplete” data refers to missing data). To combat such difficulties, dimension reduction algorithms can be employed to extract latent (i.e., underlying) patterns of exposure. Dimension reduction is premised upon the well-documented phenomenon that the variability among many correlated exposures can be faithfully expressed by substantially fewer unobserved exposure patterns.^13–17^ These patterns can reveal sources or behaviors that, if associated with adverse health outcomes, can better inform public health messaging, interventions, and policy regulations than single-chemical exposures.^7,10^

Despite this, many of the dimension reduction techniques commonly employed in environmental mixtures analyses—such as principal component analysis (PCA),^13,14,18^ factor analysis,^19,20^ non-negative matrix factorization (NMF),^21^ and clustering techniques^22–25^—were not originally developed for use in environmental health (EH) and suffer from considerable limitations; strong statistical assumptions (e.g., independent observations), sensitivity to noise and outliers, subjective pattern selection procedures, inadequate handling of missing values or values below the limit of detection (LOD), and varied software implementations all hinder robust, reproducible, and interpretable exposure pattern recognition in EH research.^10,26^ To address these challenges, we introduce pcpr, an open-source R software package implementing principal component pursuit (PCP), a robust dimension reduction technique^27–33^ specifically tailored for exposure pattern recognition in EH.^34^

PCP decomposes exposure data into a low-rank matrix, which implicitly holds (or encodes) the information needed (i.e., the mixture’s covariance structure) to identify the underlying patterns of exposure, and a sparse matrix isolating extreme or outlying exposure events^35,36^ (“extreme” and “outlying” are used interchangeably throughout the article to refer to outliers in the identification of common exposure patterns). PCP offers several advantages over conventional dimension reduction techniques: it makes minimal assumptions of exposure data, there is a single best solution to its optimization problem (rather than competing spurious solutions), it is robust to noise, extreme values are separated into the sparse matrix rather than discarded, and the number of patterns (i.e., the matrix rank) is inferred from the data, removing researcher subjectivity.

We outfitted two PCP models (presented in Zhang et al.^37^ and Cherapanamjeri et al.^38^) with four EH-specific extensions:^34^ missing-data accommodations, penalties for measurements below the LOD, non-negativity constraints, and non-convex formulations all enhance PCP’s flexibility in modeling environmental mixtures data. PCP enjoys better recovery and interpretation of latent exposure patterns over established baselines such as PCA in both simulated and applied scenarios.^34^

The open-source pcpr R package^39^ makes PCP accessible to researchers across a wide variety of disciplines and skill sets. The package is publicly available for download on CRAN, complete with EH-specific enhancements, user-friendly functions to fit and fine-tune PCP models, and extensive documentation including interactive examples with real environmental data. The functions in pcpr have already been effectively applied in several EH studies: Benavides et al.^40^ employed PCP to assess prenatal exposures and adolescent behavioral outcomes, Tao et al.^41^ used it to identify PM_2.5_ sources and their cardiovascular impacts, Wu et al.^42^ for exposome profiling of seminal plasma, and Benavides et al.^43^ to develop the Community Severance Index in New York City (NYC).

This work has two main contributions: first and foremost, we present an application-oriented introduction to the pcpr package and its many utilities, with guidance on implementation, practical examples, and a discussion of use cases and limitations. Second, we present the novel methodological enhancements that have been developed for EH-specific PCP models in the years since Gibson et al.^34^ introduced the first EH-specific PCP model, PCP-LOD. These include implementing a robust version of convex PCP^37^ and a more flexible version of non-convex PCP,^38^ both of which have been outfitted with EH-specific adaptations first presented in Gibson et al.^34^

In this article, we first provide an overview of the models available for use in pcpr, along with their EH-specific adaptations and ancillary methods for hyperparameter tuning. We then demonstrate practical use of pcpr via a real-world, fully reproducible source-apportionment study of speciated PM_2.5_ data, illustrating how pcpr can be utilized to extract interpretable patterns and outlying events amenable to downstream health modeling. The source-apportionment analysis is shipped in pcpr as a vignette, with fully reproducible R code including user-friendly access to the air pollution dataset. We conclude by discussing pcpr’s advantages, limitations, and potential for advancing mixtures research in environmental epidemiology.

## Methods

### Overview of PCP

The mixtures model assumed by PCP is formalized by the following: the observed exposure data matrix D is the sum of three unknown, latent matrices:


$$\begin{array}{c} \underset{mixture}{D_{n\times p}}=\underset{low\text{\textasciitilde{}}\text{-}rank}{L_{0}}+\underset{sparse}{S_{0}}+\underset{noise}{Z_{0}}, \end{array}$$

a low-rank matrix $L_{0}$ implicitly containing the underlying, consistent patterns of exposure, sparse corruptions $S_{0}$ identifying unique or extreme exposure events unexplained by the exposure patterns, and dense—that is, not sparse—noise $Z_{0}$ noise. All of these matrices are of dimension n×p, where n is the number of observations (e.g., study participants or measurement dates) and p is the number of exposures (chemical and/or nonchemical stressors). Beyond this observational model, the only assumptions PCP is subject to are that $L_{0}$ is not sparse, $S_{0}$ is not low-rank, and $Z_{0}\sim 𝒩\left(\mu ,\sigma^{2}\right)$ consists of i.i.d. Gaussian noise corrupting each entry in the overall exposure matrix D. Under these minimal assumptions, PCP seeks to decompose the observed mixture D into the three terms outlined in the equation above via an optimization program, thereby recovering the latent $L_{0}$ and $S_{0}$ with accurate estimates $\hat{L}$ and $\hat{S}$.

#### The low-rank matrix

The estimated low-rank matrix $\hat{L}$ provides information on the consistent exposure patterns, satisfying


$$\begin{array}{c} r=rank\left(\hat{L}\right)\ll min\left(n,p\right). \end{array}$$

The rank r corresponds to the (relatively few) number of underlying patterns governing the mixture, such as specific sources or behaviors that lead to exposure. Importantly, in convex PCP formulations, r is inferred from the observed exposure data during optimization, removing the need for the researcher to predefine the number of components (as is done in PCA or factor analysis).

$\hat{L}\in {\mathbb{R}}^{n\times p}$ remains in the original variable space, serving as a robust approximation of the true environmental mixture matrix, unperturbed by outliers (captured in $\hat{S}$) or noise (handled by PCP’s noise term). Exposure patterns can be extracted from $\hat{L}$ using matrix factorization methods (e.g., PCA, factor analysis, NMF) to generate chemical loadings and individual scores for downstream health models. This flexibility allows adaptation to mixture-specific assumptions, such as using NMF when sources are non-negative and not orthogonal or incorporating $\hat{L}$ directly into regression models depending on study design.

#### The sparse matrix

The sparse matrix $\hat{S}$ captures unusually high or low exposure events not explained by $\hat{L}$. Most entries are zero; nonzero entries—determined automatically during optimization—identify extreme exposure events. Unlike traditional methods in which researchers need to subjectively remove outliers entirely, PCP separates only the deviating portion into $\hat{S}$, preserving consistent exposure patterns in $\hat{L}$.

This distinction is critical for EH applications: for instance, PM_2.5_ potassium spikes during fireworks or wildfires can produce biased PCA-derived patterns,^30,44–46^ whereas PCP-identified patterns are made more robust by isolating such outliers into the sparse matrix $\hat{S}$. Retaining extreme events in this way allows their inclusion in health models as unique risk factors (e.g., wildfire exposure and asthma^47^) or effect modifiers (e.g., Saharan dust altering traffic-related pollution associations^48^), providing richer insight into exposure–health relationships.

### PCP algorithms

The pcpr package implements two EH-specific PCP models that are distinct from the original EH-specific PCP model, PCP-LOD^34^: the convex model *square root PCP* ($\sqrt{PCP}$)^37^ and nonconvex model *rank-based robust matrix completion* (RRMC).^38^ Their relative differences and use cases from an application perspective are summarized in Table 1. Further technical detail is given in Supplemental Sections S1 and S2, https://links.lww.com/EE/A452, for $\sqrt{PCP}$ and RRMC, respectively.

**Table 1.** The two PCP models implemented in pcpr and their relative differences and use cases

	$\sqrt{PCP}$	RRMC
pcpr package function	root_pcp()	rrmc()
Technical details	37	38
Convexity	Convex	Non-convex
Model convergence	Slower	Faster
Best suited for	Mixtures w/relatively few underlying patterns	Mixtures w/many complex underlying patterns
Use when sing(D) outputs	Rapidly decaying singular values	Slowly decaying singular values
Supports missing values?	Yes	Yes
Supports LOD penalty?	Yes	Yes
Supports non-negative output?	Yes	No
# of patterns determination	Autonomous	Semi-autonomous using the grid_search_cv() fn
Sparse event identification	Autonomous	Autonomous
Difference with PCP-LOD^34^	Less sensitive to parameter tuning	More flexible modeling of complex mixtures

### EH-specific extensions

The pcpr package is equipped with three EH-specific extensions, allowing PCP to flexibly model a variety of mixture data—without providing any additional difficulty to optimization.^34^ These modifications have been implemented in pcpr as switches that can be turned on or off depending on the needs of each environmental mixture application.

#### Accommodations for missing observations

The PCP models in pcpr reconstruct missing values according to the exposure patterns of the estimated low-rank model $\hat{L}$. This assumes that the same data-generating mechanisms govern both observed and unobserved entries. Because PCP primarily seeks accurate estimation of *patterns* rather than individual concentrations, this assumption is reasonable, but in some edge cases may not always be satisfied.

PCP can accommodate reasonable levels of random missingness with high fidelity as long as the observed correlation matrix remains representative of the underlying mixture patterns. It can also handle some systematic missingness. To the extent that the covariance structure estimated by the observed entries is not meaningfully different from the one that would have been observed had there been no missing values, then PCP is still able to recover the underlying patterns. However, PCP’s performance has only been evaluated in settings in which missingness is completely at random. Therefore, users should be cautious in situations in which missingness could be informative (missing at random and especially when missing not at random), as this may influence results and the identification of interpretable patterns. Reconstruction performance declines as missingness increases (performance also declines as noise increases, since noise can also obscure the covariance structure),^34^ and PCP cannot reconstruct entire rows or columns lacking any correlation information.

There are four main takeaways from this approach toward missing data. First, accurate recovery of missing entries depends on reliably estimating $\hat{L}$. Second, and consequently, increasing missingness degrades reconstruction of both unobserved and observed values by limiting the information available to estimate $\hat{L}$. Third, higher levels of missingness artificially inflate the sparsity of $\hat{S}$, since sparse events cannot be identified where data are absent. Finally, extending PCP to handle incomplete data enables the Monte Carlo cross-validation (MCCV) hyperparameter tuning procedure outlined in the Parameter tuning section.

#### LOD penalty

Traditionally, measurements below the LOD are imputed with fixed values such as LOD/$\sqrt{2}$,^49^ which can distort exposure distributions and bias pattern recovery.^50^ Multiple imputation reduces bias but increases computational complexity,^51^ and accommodating below-LOD values in mixtures is difficult.^52^

To address these challenges, we engineered an LOD-specific penalty $\psi_{LOD}$ that penalizes estimated observations in the PCP model differently based on their individual relationship to the researcher-provided LOD. Performance degrades as the proportion of observations falling below the LOD increases.^34^

For an in-depth dive into the mathematics of the $\psi_{LOD}$ penalty, we refer readers to Gibson et al.^34^ We also provide a summary of the LOD penalty in the Supplement (Section S4, https://links.lww.com/EE/A452).

#### Non-negativity constraint

For the $\sqrt{PCP}$ model only, we implemented an optional non-negativity constraint on $\hat{L}$ that ensures all estimated values are ≥0 non-negative, enhancing interpretability by removing negative chemical concentration estimates. This enables seamless use of methods like NMF to extract non-negative exposure patterns. For technical details, we again refer readers to Gibson et al.^34^

### Parameter tuning

Both $\sqrt{PCP}$ and RRMC rely on hyperparameters that control the sparse and noise components of the model (cf. Supplemental Sections S1 and S2, https://links.lww.com/EE/A452). Default values, based on the mixture matrix’s dimensions, are theoretically near-optimal under idealized conditions, but for real-world mixtures—corrupted by multicollinearity and noise—tuning is often needed to tailor the model to the data at hand. We developed an MCCV procedure^53^ for this purpose: it evaluates PCP’s performance across a user-defined grid of hyperparameter values by measuring recovery of repeated, randomized held-out test sets. Further detail and practical guidance are provided in the Supplement (Section S5, https://links.lww.com/EE/A452).

### Software implementation

The pcpr software is implemented as an R package^54^ and is publicly available for download on CRAN.^55^ Source code is publicly available on GitHub.^39^ The package has been published under the GPL license v3.

The pcpr package provides a comprehensive framework to facilitate exposure pattern recognition in EH research, including the following cornerstone functions:

• root_pcp(): implements $\sqrt{PCP}$ with optional EH-specific extensions (missing-data accommodations, LOD penalty, non-negativity constraint);
• rrmc(): implements RRMC with optional EH-specific extensions (missing-data accommodations, LOD penalty);
• get_pcp_defaults(): retrieves the default PCP hyperparameter values for a given data matrix D; and
• grid_search_cv(): implements the MCCV grid search procedure with optional parallelization.

The required input to these key functions is simply the exposure matrix of interest with missing values set to NA.

The pcpr package is shipped with extensive documentation, including three vignettes incorporating PCP in EH studies. The first presents a high-level, code-free overview of $\sqrt{PCP}$ and RRMC. The second uses pcpr to simulate a simple mixture with outliers, noise, and below-LOD values, before fitting and fine-tuning a PCP model benchmarked against PCA. The third employs pcpr to conduct a PCP-driven source apportionment of real-world speciated PM_2.5_ data (also shipped with the pcpr package). The methodology and results of this example source apportionment are detailed in the Application and Results sections, respectively.

### Practical considerations

Several practical considerations arise when applying PCP for exposure pattern recognition, including the pre-processing of exposure data, whether to include covariates or mixed data types, sensitivity of recovered patterns to hyperparameters, and PCP model selection. These topics are detailed in Supplemental Section S6, https://links.lww.com/EE/A452, and in pcpr package vignettes.

### Application: Air pollution source apportionment

Ambient PM_2.5_, which consists of airborne particles with a diameter of ≤2.5 μm, poses a major public health threat. It was linked to an estimated 4 million deaths worldwide annually in 2019.^56^ Understanding which sources of PM_2.5_ are most linked to health risks could help identify key targets for efficient reduction efforts.

Here we applied PCP as a pattern recognition technique to identify PM_2.5_ sources by analyzing 26 chemical species measured every 3–6 days from 1 January 2015 through 30 December 2021 in Queens, NYC. The data were obtained from the U.S. Environmental Protection Agency’s Air Quality System data mart (site ID: 36-081-0124) and are included in the pcpr package for quick and easy analysis.^57^ We used average daily concentrations for 26 PM_2.5_ constituents presented in Supplemental Table S1, https://links.lww.com/EE/A452.

Pre-processing entailed setting negative observations to zero (11.6% of the total number of observations in the data matrix) and scaling (but not centering) each chemical by dividing each measurement by the column-specific root mean square (corresponding to the standard deviation if the data were centered before being scaled).

After pre-processing, the singular values of the data matrix were visually inspected using pcpr’s sing() function (which takes in a matrix D and outputs a vector of D’s singular values) to determine which PCP model ($\sqrt{PCP}$ vs. RRMC) would be most appropriate for the analysis. Slowly decaying singular values present as a smooth graph, while sharply decaying values drop off like a sudden cliff. In this analysis, the singular values presented as slowly decaying, indicating that the nonconvex PCP algorithm RRMC, implemented in the rrmc() function, would more flexibly model the mixture’s latent data structure.

RRMC’s hyperparameters η and r were tuned using the MCCV grid search procedure implemented in pcpr’s grid_search_cv() function. Four different values for η (including the default) and ranks r=1−8 were evaluated across K=100 randomly constructed test sets, yielding 3200 model fits. The grid search was parallelized using 16 CPU cores and finished within 2 minutes on an Apple MacBook Pro with an M4 Max chip. The estimated empirically optimal parameters, $\hat{\eta }$ and $\hat{r}$, were those minimizing the mean relative test set error. The mean relative test set error is best used as a relative metric for comparing hyperparameter combinations; a value closer to 0 indicates closer adherence to the observed data. Generally, the hyperparameter combination yielding the lowest mean relative error is preferred, but this may not always be the case. In environmental mixture data, where observations are likely corrupted by noise or outliers, some departure from the observed values is expected and appropriate. A higher mean relative error may therefore reflect reasonable regularization rather than poor model performance. As such, we recommend selecting hyperparameters yielding solutions with a relatively low mean relative error, reasonable (by a priori assumptions) sparsity metrics for the recovered $\hat{S}$ matrix, and a stable rank for the recovered $\hat{L}$ matrix.

The final PCP model was then fit as $\hat{L},\hat{S}=RRMC\left(D,\hat{\eta },\hat{r}\right)$. NMF^21,58^ was fit to $\hat{L}$ (after truncating any negative values in $\hat{L}$ to 0, because RRMC lacks a non-negativity constraint) to extract chemical loadings and scores. NMF was implemented with Gaujoux and Seoighe’s^58^ R package *NMF* and optimized using the offset method by Badea.^59^ To fit the NMF model, we fixed a random seed and used 30 runs (parallelized using 16 CPU cores) to achieve stability and avoid local minima offering unstable fits. The input rank for NMF is simply the same rank r from PCP’s output $\hat{L}$ matrix. Pollution sources were interpreted from PCP–NMF loadings utilizing expert knowledge and existing literature, and the variance explained by each pattern was calculated as the sum of that pattern’s scores divided by the total sum of all pattern scores.

The data and all code for the analysis are publicly available and open-source, both within the CRAN-distributed pcpr package as a vignette and on our package’s GitHub repository.^39^

## Results

### Air pollution source apportionment

The final data matrix for the analysis included 838 observations (days) of 26 chemical species (01/01/2015–12/30/2021). The matrix was missing data for 2.2% of observations. The median percentage of missing observations for each chemical was 1.9%. No LOD information was available.

The cross-validation procedure estimated an empirically optimal rank of $\hat{r}=4$ and a regularization strength of $\hat{\eta }=0.1$. The top 10 performing hyperparameter combinations, as measured by mean relative error, are presented in Table 2.

**Table 2.** Top 10 performing RRMC hyperparameter combinations coming out of the MCCV grid search procedure, as measured by the lowest average test set relative recovery error on the Queens, NYC PM_2.5_ dataset

\newcommand\eeη $	r	Mean relative error	Mean $\hat{L}$ rank	Mean $\hat{S}$ sparsity
0.1	4	0.732	4	0.993
0.1	3	0.733	3	0.995
0.1	2	0.752	2	0.993
0.1	1	0.756	1	0.991
1.0	1	0.756	1	1.00
1.0	2	0.760	2	1.00
1.0	3	0.805	3	1.00
0.01	7	0.806	1	0.173
0.01	8	0.806	1	0.173
0.01	5	0.806	1	0.173

**Figure 1.**
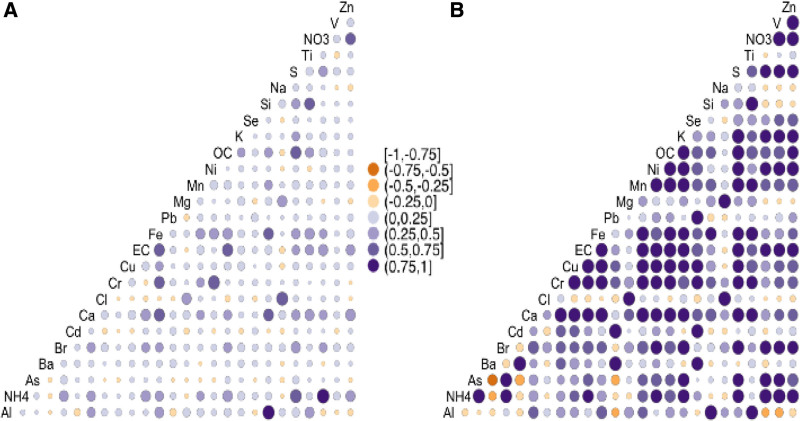
Pearson correlation coefficients among the 26 PM_2.5_ constituents of (A) the observed mixture before PCP; (B) the low-rank matrix $\hat{L}$ estimated by PCP RRMC.

Fitting the final RRMC model with $\hat{\eta }=0.1$ and $\hat{r}=4$ yielded a low-rank $\hat{L}$ matrix with four latent patterns and a sparse matrix with 99% of entries as zero and 211 entries (day-chemical species combinations) identified as extreme exposure events. Figure 1 shows the Pearson chemical correlation structure before and after running PCP via RRMC. By removing parts of extreme observations not explained by the latent patterns (i.e., outliers) as well as Gaussian noise from the observed mixture, PCP was able to pull out the underlying correlation structure governing the low-rank air pollution dataset.

**Figure 2.**
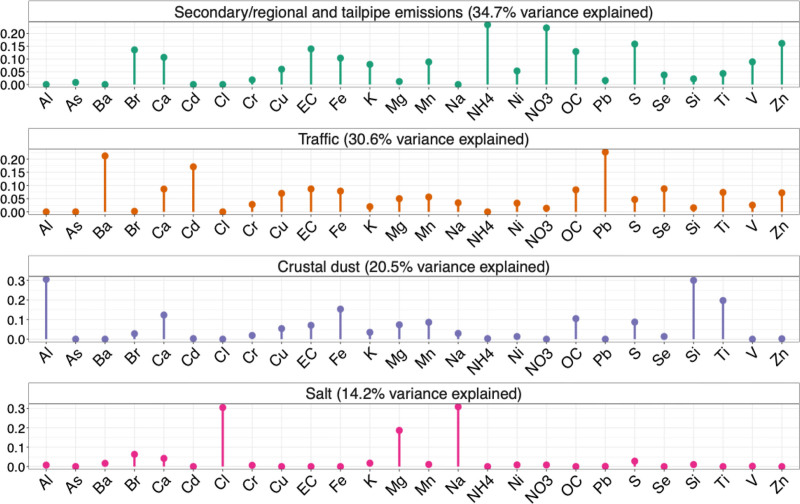
Chemical loadings for the four PCP-NMF-derived patterns: Secondary/regional mixed with tailpipe emissions, traffic, crustal dust, and salt.

After fitting NMF on $\hat{L}$, we interpreted the resulting four exposure profiles as combinations of different PM_2.5_ sources, depicted in Figure 2: (1) regional/secondary signal mixed with tailpipe emissions; (2) traffic; (3) crustal dust; and (4) salt. The regional/secondary signal mixed with tailpipe emissions was the most prominent pattern governing the mixture (explaining 34.7% of the variance in pattern scores), characterized by sulfur (S), ammonium (NH_4_), nitrate (NO_3_), organic carbon, and zinc (Zn). Traffic was next, accounting for 30.6% of the variance in the data and characterized by barium (Ba), calcium (Ca), and lead (Pb), indicating emissions from brake wear, tire wear, and construction. Crustal dust explained 20.5% of the variance in the pattern scores and was most heavily distinguished by high loadings for aluminum (Al), calcium (Ca), silicon (Si), titanium (Ti), and iron (Fe), suggesting the resuspension of crustal materials from soil and rock. Salt completed the remaining 14.2% of the mixture, marked by sodium (Na) and magnesium (Mg) chloride (Cl).

**Figure 3.**
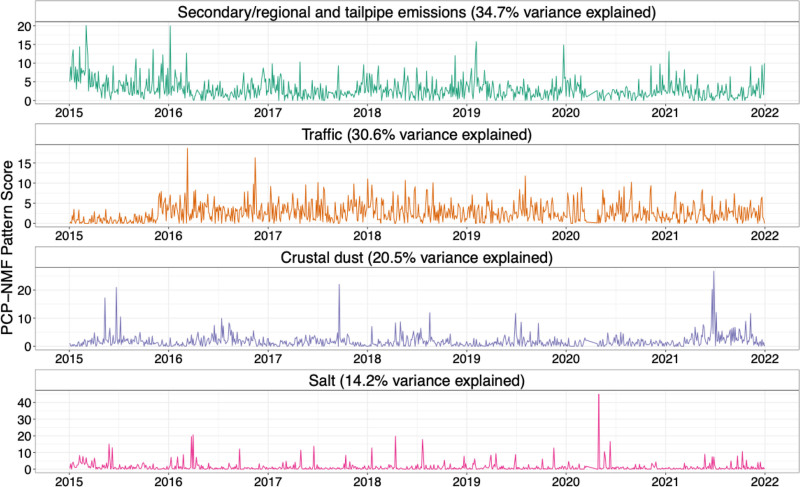
Pattern scores over time for the four PCP-NMF-derived patterns.

Figure 3 depicts the corresponding PCP-NMF-derived pattern scores over time, which can be incorporated into downstream health models. See the Discussion section for examples of techniques to include pattern scores in health models.

**Figure 4.**
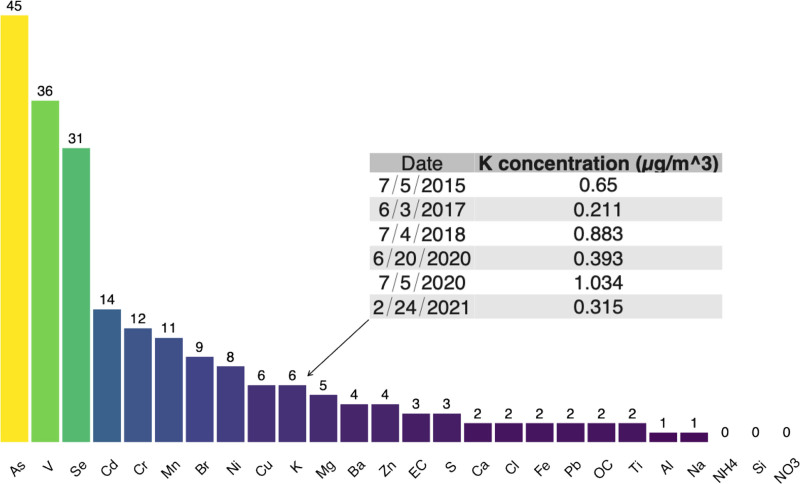
Number of extreme exposure events identified in PCP’s estimated sparse matrix $\hat{S}$ by chemical. The concentrations of the six extreme exposure events identified for potassium (K) are presented in the inset table in $\;\mu \;g/m^{3}$.

Figure 4 presents the number of outlying exposure events in $\hat{S}$ by chemical. Arsenic (As), vanadium (V), and selenium (Se) had the most outlying events. Examining individual chemicals can reveal meaningful patterns in their outliers: for instance, extreme As and Se events given in Supplemental Tables S2 and S3, https://links.lww.com/EE/A452, may indicate power plant–related activity, while extreme V concentrations given in Supplemental Table S4, https://links.lww.com/EE/A452, suggest potential residual heating oil events. Follow-up trajectory analyses could clarify these events. The inset table in Figure 4 lists extreme potassium (K) events associated with biomass burning and fireworks. PCP autonomously captured July Fourth fireworks events for each year with available data (measurements were recorded every 3–6 days, so some years do not have observations available for July Fourth or Fifth). Importantly, we emphasize that these 211 observations captured in the sparse matrix were not completely removed from $\hat{L}$; rather, only the *outlying portion* of the observations that could not be explained by the underlying consistent patterns captured in $\hat{L}$ were separated into $\hat{S}$.

## Discussion

The pcpr package provides environmental epidemiologists with an open-source, well-documented, EH-specific implementation of PCP, a dimensionality reduction method for robust pattern recognition ($\hat{L}$) and identification of extreme events ($\hat{S}$) not explained by common patterns in the exposure. Central to the pcpr package are easy-to-use functions for handling common features of environmental mixtures data, including multicollinearity, missing values, observations below the LOD, noisy or extreme measurements, and non-negativity constraints placed on the mixture matrix. Unlike traditional methods such as PCA, PCP objectively identifies the number of underlying exposure patterns (i.e., the rank) and separates outlying observations automatically, removing two subjective decision points from the analysis. In pcpr, this is accomplished by either isolating the mixture rank during optimization (as in pcpr’s implementation of convex PCP) or through the implemented data-driven MCCV grid search function (for pcpr’s nonconvex PCP), which has been outfitted with efficient parallel computing capabilities. The pcpr package comes complete with extensive documentation, including annotated example analyses utilizing both simulated and real-world data, equipping researchers with an accessible implementation of PCP that has already been deployed across a range of applications, from prenatal exposure profiling^40^ and PM_2.5_ source apportionment^41^ to exposome characterization^42^ and community-level index development.^43^

In the PM_2.5_ source-apportionment example detailed earlier (included as an R vignette in pcpr), we can see that given a high-dimensional, complex air pollution mixture suffering from missing values and high correlations among some of its chemical constituents, nonconvex PCP paired with the MCCV grid search procedure and NMF was able to extract interpretable patterns of exposure and sparse outlying exposure events. These exposure patterns and outliers can then be paired with any health outcomes of interest for robust downstream health-related or other analyses.

Take, for example, Tao et al,^41^ who applied PCP to apportion PM_2.5_ to its sources in NYC, using information across three monitoring sites, and investigated the association between source-specific PM_2.5_ and myocardial infarction (MI) hospitalizations. In this study, PCP-identified sources were carried into the health analysis through the per-day scores of the low-rank matrix: after decomposing the speciated PM_2.5_ matrix, the authors applied non-negative matrix factorization to obtain, for each source, chemical loadings (used to label sources such as traffic, crustal dust, and regional pollution) and a daily factor score quantifying that source’s contribution on each observation day. These daily scores were rescaled into interpretable, source-specific PM_2.5_ concentrations ($\;\mu \;g/m^{3}$) by regressing daily total PM_2.5_ on the scores of all sources (including a term for the summed sparse-matrix contribution to absorb sparse events) and multiplying each score by its source’s regression coefficient. The resulting daily source concentrations then were included directly as the exposure variables in a quasi-Poisson regression for daily MI admission counts, yielding effect estimates expressed as the percent change in MI admission rate per 1 $\;\mu \;g/m^{3}$ increase in each source-specific PM_2.5_.

In another study, Benavides et al.^40^ used PCP coupled with factor analysis to identify separate patterns in (1) a matrix of prenatal chemical and psychosocial exposures and (2) a matrix of adolescent behavioral symptoms. Here the linkage proceeded through per-individual factor scores: for every participant, factor analysis of the PCP-derived low-rank matrix produced a score on each identified profile (e.g., (1) prenatal exposure profiles combining air pollutants, PAH-DNA adducts, and psychosocial stressors, and (2) behavioral profiles combining attention, thought, substance use, and self-control problems). Because these scores were defined at the level of the individual, they were carried forward as standardized covariates into separate participant-level linear regressions of each behavioral profile score on each exposure profile score (adjusting for confounders), which detected a significant association between the prenatal air-pollutant-and-stress profile and a profile with high loadings of self-reported attention, thought, substance use, and self-control problems.

In both cases, the low-rank matrix yields for each unit of observation (whether a day as in Tao et al.^41^ or an individual as in Benavides et al.^40^) a score for each identified pattern, and these scores serve as the exposure variables that can be regressed against any health outcome measured on the same observational units.

There are many avenues for future work. The pcpr package is currently written in R, but core modeling functions can be made faster if they are implemented in C++ before being wrapped in R with the Rcpp package.^60^ Moreover, the optional non-negativity constraint has thus far only been implemented for convex $\sqrt{PCP}$ and remains to be developed for the nonconvex RRMC model. PCP algorithms to better recover patterns from mixture data suffering structural missingness (rather than random missingness) provide further opportunities to extend pcpr. For example, these models could recover patterns from air pollution data missing many years of observations for multiple chemicals in the mixture. PCP’s estimated sparse matrix capturing rare outliers also offers many exciting avenues for future work. Beyond outlier detection as an end goal, the sparse matrix can additionally be incorporated in downstream analyses—for instance, as a covariate capturing variation not explained by the consistent patterns, as in Tao et al,^41^ where the summed sparse contribution was included in the source-apportionment regression. Furthermore, events identified in the sparse matrix that are interpretable (e.g., the fireworks in our example analysis) could be included as separate exposure events in health models or examined as modifiers of the association between the identified patterns and the outcome of interest. We would like to note here that the events absorbed in the sparse matrix are outliers in the identification of common exposure patterns. It is possible, nonetheless, that some pattern scores are large enough to qualify as outliers in subsequent analyses.

## Conclusion

The open-source pcpr software package provides a user-friendly set of tools for robust and reproducible dimension reduction, exposure pattern recognition, and detection of extreme events not explained by identified consistent patterns tailored specifically to environmental mixtures data.

As a dimensionality reduction algorithm, PCP is rigorous in its own right: applied to a mixture matrix, it returns a denoised, low-rank version of that matrix in which consistent patterns are implicitly encoded and the influence of extreme or outlying events has been autonomously separated out. Importantly, the determination of the number of patterns governing the low-rank matrix is performed autonomously (convex PCP) or semi-autonomously (nonconvex PCP coupled with the data-driven grid search) in pcpr, eliminating the potential subjectivity that arises in more traditional methods. A key strength of PCP is that this dimensionality reduction step is decoupled from the subsequent pattern extraction step, so that the extraction technique can be chosen to confer the greatest interpretability for the problem at hand—for instance, NMF when non-negative patterns are required, PCA when orthogonal patterns are assumed, or factor analysis with an oblique rotation when correlated patterns are expected. This separation allows the dimensionality reduction to remain rigorous and reproducible across applications while the pattern extraction is tailored to the task, together yielding interpretable, reproducible, and robust patterns amenable to public health messaging and targeted interventions. Through its accompanying documentation and tutorials, pcpr makes this workflow an accessible tool for EH scientists interested in exposure pattern recognition in mixtures research.

The authors declare that they have no conflicts of interest with regard to the content of this report.

## Supplementary Material


