# Supplementary material for: Robust environmental exposure pattern recognition and outlier detection with the open-source R package pcpr
Source: Environ Epidemiol. 2026 Aug 10;10(4):e515. doi: 10.1097/EE9.0000000000000515 (PMC13461087; doi:10.1097/EE9.0000000000000515)
Supplement: Supplementary file 1 [file ee9-10-e515-s001.pdf]

SUPPLEMENTARY MATERIAL

Robust environmental exposure pattern recognition and outlier detection with the open-source R package pcpr

Lawrence G. Chillrud[a,b], Jaime Benavides[a,g], Elizabeth A. Gibson[c], Junhui Zhang[d], Jingkai Yan[e], John N. Wright[e], Jeff Goldsmith[f], Marianthi-Anna Kioumourtzoglou[a,g,h]

[a]*Department of Environmental Health Sciences, Columbia University Mailman School of Public Health, New York, NY, USA*

[b]*Department of Electrical and Computer Engineering, Northwestern University, Evanston, IL, USA*

[c]*Department of Biostatistics, Harvard T. H. Chan School of Public Health, Boston, MA, USA*

[d]*Department of Operations Research, MIT, Cambridge, MA, USA*

[e]*Department of Electrical Engineering, Columbia University, New York, NY, USA*

[f]*Department of Biostatistics, Columbia University Mailman School of Public Health, New York, NY, USA*

[g]*Department of Epidemiology, Brown University School of Public Health, Providence, RI, USA*

[h]*Institute at Brown University for Environment and Society, Providence, RI, USA*

TABLE OF CONTENTS

SUPPLEMENTARY METHODS **2**

S1: CONVEX PCP VIA $\sqrt{PCP}$ 2
S2: NON-CONVEX PCP VIA RRMC 4
S3: ACCOMMODATIONS FOR MISSING OBSERVATIONS 5
S4: THE LIMIT OF DETECTION PENALTY 5
S5: PARAMETER TUNING WITH THE MCCV GRID SEARCH PROCEDURE 6
S6: PRACTICAL CONSIDERATIONS 7

SUPPLEMENTARY TABLES **9**

S1: THE 26 CHEMICAL SPECIES FOR SOURCE APPORTIONMENT 9
S2: ARSENIC CONCENTRATIONS IN $\hat{\boldsymbol{S}}$ 10
S3: SELENIUM CONCENTRATIONS IN $\hat{\boldsymbol{S}}$ 12
S4: VANADIUM CONCENTRATIONS IN $\hat{\boldsymbol{S}}$ 13

SUPPLEMENTARY REFERENCES **15**

SUPPLEMENTARY METHODS

S1: Convex PCP via ROOT PCP

In convex PCP—the classical formulation— there is a single best solution to the optimization problem, and the rank r of $\hat{\boldsymbol{L}}$ is determined automatically during optimization, without researcher input.

These benefits come at a cost: convex PCP is computationally intensive and furthermore expects the underlying $\boldsymbol{L_0}$ matrix to possess a well-defined low-rank structure (meaning relatively few exposure patterns faithfully characterize the variation in the observed data). Well-defined low-rank structures are characterized by rapidly decaying singular values, typical of only a small subset of environmental mixtures governed by relatively few unique patterns.

The convex model available in *pcpr* is $\sqrt{PCP}$. For a comprehensive technical understanding, we refer readers to Zhang et al. (2021). Here, we detail the methodology most relevant to an EH audience.

$$\sqrt{PCP}(\boldsymbol{D}, \lambda, \mu) \rightarrow \left(\hat{\boldsymbol{L}}, \hat{\boldsymbol{S}}\right)$$

Given the observed mixture data matrix $\boldsymbol{D}$ and regularization hyperparameters λ and μ, $\sqrt{PCP}$ aims to find the best estimates $\hat{\boldsymbol{L}}$ and $\hat{\boldsymbol{S}}$ via the following objective function:

$$\sqrt{PCP} : \min_{\boldsymbol{L},\boldsymbol{S}} \ \|\boldsymbol{L}\|_* + \lambda\|\boldsymbol{S}\|_1 + \mu\|\boldsymbol{L} + \boldsymbol{S} - \boldsymbol{D}\|_F.$$

This objective function minimizes the weighted combination of three matrix norms: the nuclear norm of $\boldsymbol{L}$, denoted $\|\boldsymbol{L}\|_*$, the entry-wise ℓ_1 norm of $\boldsymbol{S}$, denoted $\|\boldsymbol{S}\|_1$, and the Frobenius norm of the noise, written as $\|\boldsymbol{L} + \boldsymbol{S} - \boldsymbol{D}\|_F$.

The nuclear norm of $\boldsymbol{L}$, often used to search for low-rank matrices, is the sum of $\boldsymbol{L}$'s singular values:

$$\|\boldsymbol{L}\|_* = \sum_{i=1}^{\min(n,p)} \sigma_i(\boldsymbol{L}).$$

Minimizing this quantity over the course of optimization incentivizes $\boldsymbol{L}$ to be low-rank by essentially shrinking the smaller singular values towards 0 (the rank r of a matrix is the same as its number of non-zero singular values σ_i).

The entry-wise ℓ_1 norm of $\boldsymbol{S}$ (also known as the "Manhattan distance") computes the sum of the absolute values of all entries in the matrix,

$$\|\boldsymbol{S}\|_1 = \sum_{i=1}^{n} \sum_{j=1}^{p} \left|\boldsymbol{S}_{ij}\right|,$$

encouraging $\boldsymbol{S}$ to be sparse when minimized (the idea being that many small entries in $\boldsymbol{S}$ should shrink to 0, leaving only a handful non-zero outlying observations). Notice that this sparsity-inducing ℓ_1 norm is scaled by the hyperparameter λ in the objective function. This allows λ to act as a knob controlling the sparsity of the output model: the greater λ is, the greater the penalization of non-zero entries in $\boldsymbol{S}$, driving

sparser $\widehat{\boldsymbol{S}}$ solutions. Candès et al. (2011) derived a universal value for λ that theoretically guarantees optimal estimation performance,

$$\lambda = \frac{1}{\sqrt{\max(n, p)}},$$

where n and p are the dimensions of the exposure matrix of interest $\boldsymbol{D} \in \mathbb{R}^{n \times p}$.

The Frobenius norm acting on the noise term $\boldsymbol{Z} = \boldsymbol{L} + \boldsymbol{S} - \boldsymbol{D}$ is the root of the sum of the square of each entry in the noise matrix:

$$\|L + S - D\|_F = \sqrt{\sum_{i=1}^{n} \sum_{j=1}^{p} \left(\boldsymbol{L}_{ij} + \boldsymbol{S}_{ij} - \boldsymbol{D}_{ij}\right)^2}.$$

Minimizing the Frobenius norm minimizes the error between the predicted and the observed values, denoising the model and ensuring that $\boldsymbol{L}$ and $\boldsymbol{S}$, taken together, have high fidelity with the original mixture data $\boldsymbol{D}$. This Frobenius term is balanced by the hyperparameter μ in the objective function, providing a dial that controls $\sqrt{PCP}$'s sensitivity to noise. The greater μ is, the more the errors between the predicted model and the observed data are penalized. To find the optimal setting of μ, previous convex PCP algorithms required knowledge of the standard deviation σ of the true noise distribution generating $\boldsymbol{Z}_0$ [Zhou et al. (2010), Chen et al. (2015), Chen et al. (2021)], which in mixtures research is almost never known *a priori*. However, for $\sqrt{PCP}$, Zhang et al. (2021) developed theory for a universal, *noise-independent* choice of μ, again based on the dimensions of the exposure matrix of interest:

$$\mu = \sqrt{\frac{\min(n, p)}{2}}.$$

Using the universal hyperparameters λ and μ as given in the equations above allow $\sqrt{PCP}$ to practically estimate low-rank and sparse pairs $(\widehat{\boldsymbol{L}}, \widehat{\boldsymbol{S}})$that are stable with respect to noise. By "stable", we mean $\sqrt{PCP}$'s reconstruction error is, in the worst-case scenario, proportional to the magnitude of the noise, $\|\boldsymbol{Z}_0\|_F$ (as opposed to less robust techniques in which reconstruction error can scale quadratically with $\|\boldsymbol{Z}_0\|_F$ in worst-case scenarios). Furthermore, Zhang et al. (2021) demonstrated that $\sqrt{PCP}$ often outperforms this upper bound in a variety of experimental settings.

None of the three terms comprising $\sqrt{PCP}$'s objective function are smooth or differentiable, making standard optimization approaches such as proximal gradient methods inapplicable. However, the objective function is convex (a single best solution exists) and separable (it is the sum of the three terms outlined above), making it amenable to optimization via the Alternating Direction Method of Multipliers (ADMM) [Boyd et al. (2011), Gao et al. (2020)]. ADMM breaks the objective function down into smaller subproblems and iteratively optimizes each by alternating from one to the other. As such, $\sqrt{PCP}$ employs the ADMM solver to minimize its objective.

S2: Non-convex PCP via RRMC

Non-convex PCP frameworks have been developed to address the limitations of convex methods. Non-convex approaches converge more rapidly and are better able to accommodate settings in which low-rank structures are less pronounced, making them better suited to modeling mixtures data.

This improved flexibility in modeling mixtures data comes with two tradeoffs. First, non-convex approaches require the researcher to specify the rank, as in PCA. This allows researchers to interrogate $\hat{\boldsymbol{L}}$ models at many different ranks, but also reintroduces the subjective issue of how to choose the optimal rank r for the model. To combat this, we developed a PCP-specific Monte Carlo cross-validation (MCCV) procedure that identifies the optimal rank using objective criteria derived from the observed data matrix (see the parameter tuning section, S4). Second, non-convex PCP runs the risk of finding spurious *local* optima, rather than the *global* optimum guaranteed by their convex counterparts. Nonetheless, recent work demonstrates that under standard theoretical assumptions, non-convex formulations can achieve performance comparable to convex PCP for well-measured mixtures [Cherapanamjeri et al. (2017), Netrapalli et al. (2014), Chen et al. (2021), Ge et al. (2017), Yi et al. (2016)]. In practical settings that can often violate theoretical conditions, e.g. for messier mixtures suffering non-random missingness, non-convex PCP's theoretical lower performance bound loosens, however it often outperforms this empirically. These developments provide strong justification for applying non-convex PCP in environmental mixtures research.

RRMC is the non-convex model shipped with *pcpr*. We refer readers to Cherapanamjeri et al. (2017) for full mathematical details; below we outline the methodology most relevant to an EH audience.

$$RRMC(\boldsymbol{D}, r, \eta) \to \left(\hat{\boldsymbol{L}}^{(1):(r)}, \hat{\boldsymbol{S}}^{(1):(r)}\right)$$

Given the observed mixture data matrix $\boldsymbol{D}$, desired rank r, and regularization hyperparameter η, RRMC seeks to find the best estimates $\hat{\boldsymbol{L}}$ and $\hat{\boldsymbol{S}}$ using an incremental rank-based PCP strategy. That is, RRMC will first estimate a rank 1 model $\left(\hat{\boldsymbol{L}}^{(1)}, \hat{\boldsymbol{S}}^{(1)}\right)$, before using that as the initialization point to construct a rank 2 model $\left(\hat{\boldsymbol{L}}^{(2)}, \hat{\boldsymbol{S}}^{(2)}\right)$, and so on, until the desired rank r model $\left(\hat{\boldsymbol{L}}^{(r)}, \hat{\boldsymbol{S}}^{(r)}\right)$ is recovered. All models from ranks 1 through r are returned by RRMC in this way. This incremental rank-based scheme is achieved via the following objective function:

$$RRMC: \min_{\boldsymbol{L},\boldsymbol{S}} \mathfrak{T}_{\text{rank}(\boldsymbol{L})\leq r} + \eta\|\boldsymbol{S}\|_0 + \|\boldsymbol{L}+\boldsymbol{S}-\boldsymbol{D}\|_F^2.$$

This objective function minimizes the weighted combination of three terms: a characteristic function checking whether $\boldsymbol{L}$ is strictly of rank r or not, denoted $\mathfrak{T}_{\text{rank}(\boldsymbol{L})\leq r}$ the ℓ_0 norm of $\boldsymbol{S}$, denoted $\|\boldsymbol{S}\|_0$, and the squared Frobenius norm of the noise, written as $\|\boldsymbol{L}+\boldsymbol{S}-\boldsymbol{D}\|_F^2$.

In RRMC, the characteristic function handles recovery of the $\boldsymbol{L}$ matrix, replacing the nuclear norm from $\sqrt{PCP}$. Formally,

$$\mathfrak{T}_{\text{rank}(\boldsymbol{L})\leq r} = \begin{cases} 0, & \text{if rank}(\boldsymbol{L}) \leq r \\ +\infty, & otherwise \end{cases}$$

To satisfy the characteristic function during optimization at stage k of RRMC's incremental procedure–that is, when RRMC is estimating the rank k model $\left(\hat{\boldsymbol{L}}^{(k)}, \hat{\boldsymbol{S}}^{(k)}\right)$–projected gradient descent is used to

project $\boldsymbol{L}$ onto the space of all rank k matrices. This can be efficiently done using a singular value decomposition (SVD) of $\boldsymbol{L}$.

The ℓ_0 norm of $\boldsymbol{S}$ is RRMC's analog of the ℓ_1 norm in $\sqrt{PCP}$. The ℓ_0 norm (technically not a norm, but rather a convienent abuse of notation) simply counts the number of non-zero entries in $\boldsymbol{S}$. To minimize this ℓ_0 metric, an element-wise hard-thresholding operator $\mathcal{HT}_\zeta$ with threshold ζ is employed:

$$\mathcal{HT}_\zeta(\boldsymbol{S}_{ij}) = \begin{cases} \boldsymbol{S}_{ij}, & \text{if } |\boldsymbol{S}_{ij}| \geq \zeta \\ 0, & otherwise \end{cases}$$

Over the course of optimization, this hard-thresholding operator $\mathcal{HT}_\zeta$ subjects each entry in the residual $\boldsymbol{L} - \boldsymbol{D}$ to a threshold ζ (determined using the hyperparameter η), thereby iteratively fashioning the sparse matrix $\boldsymbol{S}$. Entries in the residual whose absolute values are below the calculated threshold ζ are set to 0, otherwise these residuals are deemed sparse events, and populate the current $\widehat{\boldsymbol{S}}$ iteration. Importantly, ζ is an adaptive threshold, meaning the value of ζ changes with each iteration of optimization, adapting to the current stage of the rank-based procedure.

Scaling both the ℓ_0 norm and the adaptive threshold value ζ, the hyperparameter η is the knob controlling sparsity in RRMC. A larger η incurs greater penalization of non-zero entries in $\boldsymbol{S}$, as well as a higher threshold ζ. This drives more entries in $\boldsymbol{S}$ to be set to 0 and ultimately yields a sparser solution. Intuitively, because η is the only hyperparameter balancing the terms in RRMC's objective, it can loosely be thought of as a ratio between $\sqrt{\text{PCP}}$'s λ and μ. In the *pcpr* package, the default value of $\eta = \lambda/\mu$, where λ and μ are set to their theoretical optimal values given in the above section **S1**.

The squared Frobenius norm of the noise term in RRMC's objective function serves the same purpose as the Frobenius norm in $\sqrt{\text{PCP}}$.

S3: Accommodations for missing observations

The PCP models in *pcpr* handle missing values in the exposure data matrix $\boldsymbol{D}$ by applying the penalties defined by their objective functions *only to those entries that are actually observed*, consistent with the literature on robust matrix completion [Chen et al. (2013), Li et al. (2013)]. Formally, given a ground-truth exposure matrix $\boldsymbol{D}_{n\times p}$, whose observed entries are in the set $\Omega \subseteq [n] \times [p]$, the observed exposure matrix can be written as $\mathcal{P}_\Omega(\boldsymbol{D})$, where $\mathcal{P}_\Omega$ is a sampling operator preserving only the entries in $\boldsymbol{D}$ that belong to the observed set Ω. The PCP objective functions (given in supplementary methods sections S1 and S2) can be rewritten using this notation to model partially-observed mixtures data.

S4: The limit of detection penalty

PCP's limit of detection (LOD) penalty, ψ_{LOD}, treats any estimations of values known to be below the LOD as equally valid if their approximations fall between 0 and the LOD. Over the course of optimization, observations below the LOD are pushed into this known range $[0, LOD]$using penalties from above and below: should a $< LOD$ estimate be < 0, it is stringently penalized, since measured concentrations cannot be negative. On the other hand, if a $< LOD$ estimate is $> LOD$, it is also penalized: less so than when < 0, but more so than observations known to be above the LOD, because we have prior information that these observations must be below LOD. Observations known to be above the LOD are penalized as usual, using the Frobenius norms in the original objective functions.

Our prior work demonstrated that in simulated settings with up to 50% of the data below the LOD, PCP equipped with ψ_{LOD} boasts superior accuracy of recovered $\hat{\boldsymbol{L}}$ models compared to PCA coupled with $\frac{LOD}{\sqrt{2}}$ imputation [Gibson et al. (2022)]. PCP even outperforms PCA in low-noise scenarios with as much as 75% of the data corrupted below the LOD. In situations in which we simulated extreme noise in the mixture and large proportion of the total number of observations below the LOD (i.e., $> 75\%$), both PCA and PCP performed poorly.

To utilize the ψ_{LOD} penalty in the *pcpr* package, the analyst may specify the LOD either as a scalar (which would signify a universal LOD common across all chemicals and individuals in the mixture), a vector (meaning the LOD is chemical-specific, with each entry in the LOD vector corresponding to an LOD for each column, i.e., chemical, in the mixture matrix) or a matrix (indicating that each entry in the mixture matrix has its own unique LOD, therefore the LOD is both chemical- and individual-specific).

S5: Parameter tuning with the MCCV grid search procedure

While the universal values for hyperparameters λ and μ guarantee near-optimal estimation performance from $\sqrt{PCP}$, they do so in theoretical settings where the target (i.e., ground-truth) low-rank matrix $\boldsymbol{L_0}$ can be accurately modelled as low-rank. Environmental mixtures data, however, usually can only be *approximately* modelled as low-rank, due to the complexity of the data and large volume of noise obscuring the latent low-rank structure. Consequently, it is often necessary to fine-tune λ and μ when applying $\sqrt{PCP}$ to some observed exposure matrix $\boldsymbol{D}$. The same reasoning applies to η when applying RRMC. Of additional importance when applying RRMC is objectively determining a rank r to use for the estimated model, as RRMC's non-convex framework requires r to be specified by the researcher.

As such, we have developed a PCP-specific Monte Carlo-style cross-validation (MCCV) procedure to help tune $\sqrt{PCP}$'s hyperparameters λ and μ, and RRMC's hyperparameters η and r. First, the researcher must define a grid $\boldsymbol{G}$ of hyperparameters $\overrightarrow{g_m}$ to search through, using the optimal or default parameters as starting points and expanding out. Each unique setting of hyperparameters is given by $\overrightarrow{g_m} \in \boldsymbol{G}$, where $\overrightarrow{g_m} = [\lambda_m, \mu_m]$ in the case of $\sqrt{PCP}$ or $\overrightarrow{g_m} = [\eta_m, r_m]$ in the case of RRMC.

Then, given a PCP function f (where $f = \sqrt{PCP}$ or RRMC), the grid $\boldsymbol{G}$, the exposure matrix of interest $\boldsymbol{D}$, a corruption percentage ξ, and number of runs K, the following MCCV procedure is implemented for each unique parameter setting $\overrightarrow{g_m} \in \boldsymbol{G}$:

1. Randomly corrupt ξ % of the observed exposure data $\boldsymbol{D}$ as missing (`NA` in R) to serve as a held-out test set, denoted Ω^c. This yields the corrupted test matrix $\widetilde{\boldsymbol{D}} = P_\Omega(\boldsymbol{D})$ with an observed training set of entries Ω.
2. Using the current hyperparameters $\overrightarrow{g_m}$, run the PCP algorithm of choice f on the test matrix $\widetilde{\boldsymbol{D}}$ to obtain $\hat{\boldsymbol{L}}$ and $\hat{\boldsymbol{S}}$, i.e. $\hat{\boldsymbol{L}}, \hat{\boldsymbol{S}} = f\left(\widetilde{\boldsymbol{D}}, \overrightarrow{g_m}\right)$.
3. Record the relative recovery error of those entries in the held-out test set Ω^c,
$$\frac{\left\|P_{\Omega^c}(\boldsymbol{D} - \hat{\boldsymbol{L}})\right\|_F}{\|P_{\Omega^c}(\boldsymbol{D})\|_F},$$
as well as other useful metadata regarding the run, such as the rank of the output $\hat{\boldsymbol{L}}$ matrix, the sparsity of the output $\hat{\boldsymbol{S}}$ matrix, and the number of iterations the run took to converge.
4. Repeat steps 1-3 a total of K-many times, each time using a new randomly corrupted test set Ω^c.

5. Average the relative recovery errors—along with the returned rank, sparsity, and convergence statistics—of the K-many runs. These averaged statistics are the metrics ascribed to the current hyperparameter setting $\overrightarrow{g_m}$ being evaluated in $\boldsymbol{G}$.

For the cross-validated grid search, both ξ and K are specified by the researcher. In the *pcpr* package, the default values for these settings are $\xi = 0.05$ and $K = 100$, meaning each hyperparameter setting in $\boldsymbol{G}$ is tested 100 times, with each test set Ω^c comprising 5% of the observed entries in $\boldsymbol{D}$.

Keeping ξ relatively low (e.g., between 2 and 8%) encourages the test matrix $\widetilde{\boldsymbol{D}}$ to faithfully represent the observed matrix $\boldsymbol{D}$. This ensures that the problems facing PCP in the grid search closely resemble the actual problem PCP will solve during analysis, once hyperparameters have been tuned. If ξ is too large (e.g., 25% or higher), then the grid search is effectively modeling a matrix completion problem, instead of the desired low-rank and sparse decomposition problem.

Choosing a reasonable value for K is dependent on this need to keep ξ relatively small. Ideally, a large enough K is used so that many (if not all) of the entries in $\boldsymbol{D}$ are likely to eventually be tested. Note that since entries in Ω^c are chosen *randomly* for all tests 1 through K, in the worst-case scenario, the same exact test set could be drawn each time. In the best-case scenario, a different test set is obtained every time, providing balanced coverage of $\boldsymbol{D}$. Typically, overlapping test sets will be chosen that slightly oversample some entries in $\boldsymbol{D}$ and slightly under sample others. With a sufficiently large K, balanced coverage of all entries in $\boldsymbol{D}$ can be achieved.

Once the grid search of $\boldsymbol{G}$ has been conducted, the optimal hyperparameters can be chosen by examining the output statistics for each hyperparameter setting in $\boldsymbol{G}$. Hyperparameters that fail to converge can be discarded, as well as those with unreasonable sparsity or rank measurements (unreasonable meaning these reported metrics are not corroborated by prior assumptions, knowledge, or work). The hyperparameter setting with the lowest relative error among the remaining feasible options may then be selected and used in analysis.

S6: Practical considerations

Several practical considerations arise when applying PCP for exposure pattern recognition.

First, PCP requires continuous exposure variables, as does PCA, NMF, and factor analysis. Applying PCP to mixed data types (e.g., a combination of continuous and binary variables) would conflate true latent structure with artifactual variation driven by variable type. Extensions of PCP to mixed data remain an area for future work.

Next, covariates beyond the exposures of interest (e.g., weather variables, SES-related factors) can be included in PCP, and may be appropriate depending on the scientific question and the intended downstream use for the identified patterns. However, covariate inclusion should be carefully considered. Including additional variables will influence the estimated patterns and may reduce interpretability. For example, including weather covariates in an air pollution mixture will yield patterns that reflect a mix of pollution sources as well as weather patterns, rather than pollution sources alone. Furthermore, when confounders from a subsequent health analysis are included in the PCP step, the derived patterns may become more strongly associated with those confounders, making downstream confounding adjustment more sensitive to model misspecification and increasing the risk of residual confounding. As such, the inclusion of covariates should be carefully considered with respect to both interpretability of the derived patterns and implications for subsequent health-effect estimation.

Because chemical concentrations are observed in varying units of measurement, with differing numeric scales and variances, some pre-processing of the exposure data is encouraged before applying PCP models. While the optimization theory underpinning PCP does not require any assumptions for the observed data matrix $\boldsymbol{D}$, in practice we find that PCP converges much more rapidly when the data have been standardized in some way (e.g., scaling chemical concentrations column-wise by their standard deviations or column-wise min-max scaling). Therefore, we recommend employing pre-processing techniques that can standardize chemicals in $\boldsymbol{D}$ without meaningfully altering the shape of the original chemical distributions nor the underlying correlation matrix governing the data.

It is important to keep in mind that PCP is after accurate recovery of latent *patterns*, rather than accurate recovery of individual chemical concentrations. As such, it is good practice to evaluate the sensitivity of identified patterns to choices in PCP hyperparameters (especially RRMC's rank r) that produce similarly performing yet quantitatively different results in the grid search.

Finally, when deciding whether to utilize $\sqrt{PCP}$ or RRMC in analyses, we recommend exploring the structure of the mixture of interest. Because $\sqrt{PCP}$ is convex, it is well-suited for data possessing a strong low-rank structure characterized by rapidly decaying singular values. RRMC can better model mixtures with slowly decaying singular values where the rank is obscured by noise. Prior knowledge and clearly stated assumptions about the number of expected patterns contributing to the mixture can aid in this decision as well, as $\sqrt{PCP}$ tends to prefer lower-rank models compared with RRMC.

Additional guidance is provided in the *pcpr* package vignettes.

SUPPLEMENTARY TABLES

Table S1: The 26 chemical species of PM2.5 used in the source apportionment analysis and their abbreviations.

Chemical species	Abbr.
Aluminum	Al
Ammonium	NH_4
Arsenic	As
Barium	Ba
Bromine	Br
Cadmium	Cd
Calcium	Ca
Chlorine	Cl
Elemental carbon	EC
Organic carbon	OC
Chromium	Cr
Copper	Cu
Iron	Fe
Lead	Pb
Magnesium	Mg
Manganese	Mn
Nickel	Ni
Potassium	K
Selenium	Se
Silicon	Si
Sodium	Na
Sulfur	S
Titanium	Ti
Nitrate	NO_3
Vanadium	V
Zinc	Zn

Table S2: The 45 extreme arsenic (As) concentrations (in µg/m^3) identified in PCP's estimated sparse matrix $\hat{\boldsymbol{S}}$ and their date of observation.

As (µg/m^3)	Date
0.002	02/17/2015
0.003	03/10/2015
0.003	05/24/2015
0.002	06/11/2015
0.002	06/26/2015
0.002	07/08/2015
0.002	07/17/2015
0.002	07/20/2015
0.002	08/16/2015
0.002	09/27/2015
0.002	10/03/2015
0.002	11/11/2015
0.003	11/20/2015
0.004	12/05/2015
0.004	12/26/2015
0.002	02/18/2016
0.003	02/27/2016
0.002	03/01/2016
0.005	03/07/2016
0.002	03/22/2016
0.002	04/09/2016
0.005	04/12/2016
0.004	05/24/2016
0.004	05/27/2016
0.004	06/02/2016
0.006	07/23/2016
0.003	07/26/2016
0.006	08/07/2016
0.002	08/16/2016
0.004	09/09/2016
0.002	09/15/2016
0.004	11/20/2016
0.003	01/16/2017
0.002	06/15/2017

0.003	06/27/2017
0.002	09/19/2017
0.004	12/15/2017
0.005	02/16/2018
0.003	03/03/2018
0.002	03/27/2018
0.003	04/08/2018
0.005	06/25/2018
0.006	07/01/2018
0.003	08/12/2018
0.005	08/27/2018

Table S3: The 31 extreme selenium (Se) concentrations (in μg/m^3) identified in PCP's estimated sparse matrix $\widehat{\boldsymbol{S}}$ and their date of observation.

Se (μg/m^3)	Date
0.003	12/20/2015
0.005	01/10/2016
0.006	01/22/2016
0.004	02/03/2016
0.004	03/01/2016
0.003	08/22/2016
0.003	09/15/2016
0.005	10/27/2016
0.003	10/30/2016
0.004	11/20/2016
0.004	12/23/2016
0.003	01/10/2017
0.004	01/31/2017
0.003	04/04/2017
0.004	05/19/2017
0.003	06/24/2017
0.004	01/14/2018
0.004	02/25/2018
0.004	05/14/2018
0.003	05/23/2018
0.003	06/22/2018
0.004	08/21/2018
0.003	01/09/2019
0.003	01/18/2019
0.003	02/11/2019
0.003	08/22/2019
0.004	10/18/2019
0.005	05/12/2020
0.003	05/15/2020
0.003	07/17/2020
0.004	02/21/2021

Table S4: The 36 extreme vanadium (V) concentrations (in µg/m³) identified in PCP's estimated sparse matrix $\widehat{\boldsymbol{S}}$ and their date of observation.

V (µg/m³)	Date
0.002	01/06/2015
0.002	01/15/2015
0.003	03/01/2015
0.002	03/16/2015
0.003	05/06/2015
0.002	08/10/2015
0.002	01/04/2016
0.003	01/13/2016
0.003	02/24/2016
0.002	03/13/2016
0.003	03/25/2016
0.002	04/03/2016
0.002	04/24/2016
0.002	05/09/2016
0.002	05/15/2016
0.002	05/21/2016
0.002	06/23/2016
0.002	07/14/2016
0.003	11/26/2016
0.002	11/29/2016
0.002	01/13/2017
0.003	05/10/2017
0.002	07/15/2017
0.002	10/01/2017
0.002	10/07/2017
0.002	10/28/2017
0.002	11/21/2017
0.003	05/26/2018
0.002	06/10/2018
0.002	06/25/2018
0.002	07/28/2018
0.002	10/14/2018
0.002	01/12/2019
0.002	02/20/2019

0.002	05/15/2019
0.002	07/21/2021

REFERENCES

[Boyd et al. (2011)] S. Boyd, N. Parikh, E. Chu, B. Peleato, J. Eckstein, et al., Distributed optimization and statistical learning via the alternating direction method of multipliers, Foundations and Trends in Machine learning 3 (2011) 1–122.

[Candès et al. (2011)] E. J. Candès, X. Li, Y. Ma, J. Wright, Robust principal component analysis?, Journal of the ACM (JACM) 58 (2011) 1–37.

[Chen et al. (2013)] Y. Chen, A. Jalali, S. Sanghavi, C. Caramanis, Low-rank matrix recovery from errors and erasures, IEEE Transactions on Information Theory 59 (2013) 4324–4337.

[Chen et al. (2015)] Y. Chen, M. J. Wainwright, Fast low-rank estimation by projected gradient descent: General statistical and algorithmic guarantees, arXiv preprint arXiv:1509.03025 (2015)

[Chen et al. (2021)] Y. Chen, J. Fan, C. Ma, Y. Yan, Bridging convex and nonconvex optimization in robust pca: Noise, outliers and missing data, The Annals of Statistics 49 (2021) 2948–2971

[Cherapanamjeri (2017)] Y. Cherapanamjeri, K. Gupta, P. Jain, Nearly optimal robust matrix completion, in: International Conference on Machine Learning, PMLR, 2017, pp. 797–805.

[Gao et al. (2020)] W. Gao, D. Goldfarb, F. E. Curtis, Admm for multiaffine constrained optimization, Optimization Methods and Software 35 (2020) 257–303.

[Ge et al. (2017)] R. Ge, C. Jin, Y. Zheng, No spurious local minima in nonconvex low rank problems: A unified geometric analysis, in: International Conference on Machine Learning, PMLR, 2017, pp. 1233–1242.

[Gibson et al. (2022)] E. A. Gibson, J. Zhang, J. Yan, L. Chillrud, J. Benavides, Y. Nunez, J. B. Herbstman, J. Goldsmith, J. Wright, M.-A. Kioumourtzoglou, Principal component pursuit for pattern identification in environmental mixtures, Environmental health perspectives 130 (2022) 117008.

[Li et al. (2013)] X. Li, Compressed sensing and matrix completion with constant proportion of corruptions, Constructive Approximation 37 (2013) 73–99.

[Netrapalli et al. (2014)] P. Netrapalli, N. UN, S. Sanghavi, A. Anandkumar, P. Jain, Non-convex robust pca, Advances in Neural Information Processing Systems 27 (2014).

[Yi et al. (2016)] X. Yi, D. Park, Y. Chen, C. Caramanis, Fast algorithms for robust pca via gradient descent, Advances in neural information processing systems 29 (2016).

[Zhang et al. (2021)] J. Zhang, J. Yan, J. Wright, Square root principal component pursuit: Tuning-free noisy robust matrix recovery, Advances in Neural Information Processing Systems 34 (2021) 29464–29475.

[Zhou et al. (2010)] Z. Zhou, X. Li, J. Wright, E. Candes, Y. Ma, Stable principal component pursuit, in: 2010 IEEE international symposium on information theory, IEEE, 2010, pp. 1518–1522.
